# Use of multiple recreational drugs is associated with new HIV infections among men who have sex with men in China: a multicenter cross-sectional survey

**DOI:** 10.1186/s12889-021-10223-y

**Published:** 2021-02-15

**Authors:** Xiang Mao, Sequoia I. Leuba, Qinghai Hu, Hongjing Yan, Zhe Wang, Lin Lu, Minghua Zhuang, Xi Chen, Jihua Fu, Wenqing Geng, Yongjun Jiang, Hong Shang, Junjie Xu

**Affiliations:** 1 NHC Key Laboratory of AIDS Immunology (China Medical University), National Clinical Research Center for Laboratory Medicine, The First Affiliated Hospital of China Medical University, No 155, Nanjing North Street, Heping District, Shenyang, 110001 Liaoning Province China; 2 Key Laboratory of AIDS Immunology, Chinese Academy of Medical Sciences, Shenyang, 110001 China; 3 Key Laboratory of AIDS Immunology of Liaoning Province, Shenyang, 110001 China; 4 Collaborative Innovation Center for Diagnosis and Treatment of Infectious Diseases, 79 Qingchun Street, Hangzhou, 310003 China; 5 Department of Epidemiology, University of North Carolina at Chapel Hill, NC Chapel Hill, USA; 6 Jiangsu Provincial Centers for Disease Control and Prevention, Nanjing, 210009 China; 7 He’nan Provincial Centers for Disease Control and Prevention, Zhengzhou, 450022 China; 8 Yunnan Provincial Centers for Disease Control and Prevention, Kunming, 650022 China; 9 Shanghai Municipal Centers for Disease Control and Prevention, Shanghai, 200336 China; 10 Hu’nan Provincial Centers for Disease Control and Prevention, Changsha, 410005 China; 11 Shandong Provincial Centers for Disease Control and Prevention, Jinan, 250014 China

**Keywords:** Recreational drug use, multiple recreation drug use, Chinese MSM, Recent HIV infection, HIV-related high-risk behaviors

## Abstract

**Background:**

There is limited information about the types of recreational drugs used by men who have sex with men (MSM) in China or the consequent impact on sexual health and human immunodeficiency virus (HIV) acquisition.

**Methods:**

We recruited MSM from seven cities in China between 2012 and 2013 using multiple approaches including advertisements on gay websites, collaborating with local MSM community-based organizations, peer referrals, and venues such as gay bars and bathrooms visited by MSM. We divided participants into four subgroups based on the number of recreational drugs (RDs) used in the previous 6 months. We defined use of multiple RDs as use of ≥2 types of RDs. Demographics and HIV-related high-risk behaviors were collected, and blood samples were tested for recent HIV infection by the HIV-1 subtypes B, E, and D immunoglobulin G capture enzyme immunoassay (BED-CEIA). We used multivariable logistic regression adjusted for sociodemographics to determine the adjusted odds ratios (aORs) and associated 95% confidence intervals (CIs) of the subgroups of RD use for recent or established HIV infection.

**Results:**

A total of 4496 Chinese MSM participated; 28.4% used RDs, and 5% used multiple types of RDs. The prevalence of each RD use was as follows: poppers (25.9%), ecstasy (2.4%), ketamine (1.2%), amphetamine (0.6%), tramadol (0.4%), methamphetamine (3.8%), and codeine (1.9%). Users of multiple RDs commonly used poppers combined with one or more other types of RDs. Multiple RD users were likely to be aged 26–30 years (vs. 18–25 and > 30 years), live in non-local cities (vs. local cities), never married (vs. married), have a high monthly income (vs. no income and 1–599 USD), use versatile positions during anal intercourse (vs. top or bottom), and have inadequate HIV-related prevention knowledge (vs. adequate). As the number of RDs used in the previous 6 months increased, the prevalence of HIV-related high-risk behaviors increased (*P* < 0.05 for all). The odds of recent HIV infection were higher among those who used one type (aOR = 2.2, 95% CI: 1.5–3.0) or two types of RD (aOR=2.3, 95% CI: 1.0-5.2) in the previous 6 months compared to the odds among those who did not use RDs.

**Conclusion:**

The level and pattern of multiple RD use among Chinese MSM were different from high-income countries. MSM who used more RDs are more likely to engage in high-risk sexual behaviors, and these behaviors may be associated with increases in new HIV infections.

**Supplementary Information:**

The online version contains supplementary material available at 10.1186/s12889-021-10223-y.

## Background

Men who have sex with men (MSM) experience a disproportionately high burden of human immunodeficiency virus (HIV) infection [1]. The use of recreational drugs (RDs) among this population is a severe public health concern in China and elsewhere [2–4]. Unlike traditional drugs such as heroin and opium, ecstasy and methamphetamine are stimulants synthesized in laboratories. Most RDs have hallucinogenic effects and are associated with psychological dependence. RDs are challenging to quit, and excessive ingestion leads to severe consequences such as sudden death. Therefore, RDs are expressly prohibited in China [5]. At the end of the twentieth century, RD use was widespread among MSM in high-income countries [6]. This phenomenon is currently expanding to MSM communities in China and other low-to-middle income countries [3, 7–9]. RDs can enhance sexual function [10], increase sexual pleasure [11], and decrease pain during anal intercourse [12]. However, RD use has been reported to correlate with high-risk sexual behaviors and increased risk of HIV infection [11–13].

In this context, use of multiple RDs refers to using several RDs simultaneously or during the same period to achieve a particular effect. Use of multiple RDs and its correlation with HIV infection has attracted worldwide attention. Use of multiple drugs compared to use of a single drug is more likely to be associated with the transmission of HIV or other sexually transmitted infections (STIs) [14]. The use of multiple RDs alters the user’s mental state and decreases cognitive inhibition [15]. This behavior leads to an increased prevalence of condomless sex (CLS), CLS with HIV-positive partners, group sex, and increased numbers of sexual partners among MSM [16, 17]. Compared with using a single RD, any combination of psychoactive drugs (e.g., methamphetamine, ecstasy) and physiologically active substances (e.g., poppers, erectile-dysfunction agents) can increase the risk of HIV acquisition dramatically [18].

Despite the severity of this problem, most research on the use of multiple RDs has been performed in high-income countries [2, 17]. Because of political, legal, and cultural differences, these studies’ results may be generalizable to low-to-middle income countries [8, 19]. Furthermore, HIV incidence among MSM is higher in low-to-middle income countries than in high-income countries [20], suggesting a need to study the effects of RD use on recent HIV infection in low-to-middle income countries.

China is the largest low-to-middle income country in the world. The HIV prevalence among Chinese MSM was 6.9% in 2019 [1, 21]. By the end of 2017, there were 2.6 million drug addicts nationwide [22]. The RD use rate among Chinese MSM ranged from 24.1 to 77.1% [23–25]. Thus, it is imperative to understand the relationship between the use of multiple RDs and recent HIV infection among Chinese MSM to develop tailored interventions that limit RD use, specifically in the hopes of mitigating the HIV epidemic. The present study aims to provide researchers and clinicians with information about patterns of use of RDs among Chinese MSM and illustrate the impact of use of multiple RDs on HIV-related high-risk behaviors and HIV acquisition.

## Methods

### Study participants and questionnaire

To better understand the HIV epidemic among Chinese MSM, we conducted a multicenter cross-sectional survey among Chinese MSM in seven large cities in China (Shenyang, Ji’nan, Zhengzhou, Shanghai, Nanjing, Changsha, and Kunming) from June 2012 to June 2013. The Cruising areas and service points for MSM were used as the sampling sites. Site-specific sampling periods were determined based on attendance and hours of operation. We used several approaches to recruit participants: advertisements on gay websites, collaboration with local MSM community-based organizations, peer referrals, and venues such as gay bars and bathrooms visited by MSM. To be eligible for this study, participants had to be born male, aged 16 years or older, self-reported having anal/oral sex experiences with a male within the last year, and able to provide informed consent.

Eligible participants completed an anonymous structured questionnaire concerning sociodemographics, recent sexual behaviors, and other HIV-related high-risk behaviors: (1) demographics: age, residence, city, education, occupation, marital status, monthly income (USD), and predominant sex position in anal intercourse (AI); (2) recent sexual behaviors or HIV-related high-risk behaviors: age of sexual debut with males, the main venue of seeking male sexual partners in the previous 6 months, group sex in the previous 6 months, number of male sexual partners in the previous 6 months, commercial sex in the previous 6 months, mucosally traumatic sex in the previous 6 months, condom breaks during AI in the previous 6 months, STI-related symptoms in the previous year, and non-Chinese male sexual partners in the previous 6 months (Additional file 1).

Nine relevant questions assessed knowledge of prevention of HIV infection. If the participant answered all questions correctly, we defined them as having an “adequate” knowledge towards the prevention of HIV infection. The questions were as follows: (1) Is it possible for a person who looks healthy to carry HIV? (2) Is it possible to be infected with HIV through transfusion of blood or blood products with HIV? (3) Is it possible to be infected through sharing needles with HIV-infected persons or Acquired Immunodeficiency Syndrome (AIDS) patients? (4) Can the proper use of condoms in each sexual activity reduce the risk of HIV transmission? (5) Can having sex with only a single HIV-uninfected sexual partner reduce the risk of HIV transmission? (6) Can an HIV-infected pregnant woman transmit HIV to her child? (7) Is it possible to be infected through eating with HIV-infected persons or AIDS patients? (8) Is it possible to be infected through mosquito bites? and (9) If you know or suspect that your partner has AIDS, will you stop having sex with him?

Participants were asked about their use (nonmedical or recreational) of seven commonly used RDs at parties or during sexual contact in the previous 6 months: poppers, ecstasy, methamphetamine, amphetamine, codeine, tramadol, and ketamine. We grouped participants based on the number of drugs used in the previous 6 months (i.e., no drug, single drug (1DUs), two types of drugs (2DUs), and ≥ 3 types of drugs (3DUs)). Use of multiple RDs was defined as use of ≥2 types of drugs in the previous 6 months.

### Laboratory testing

**Fig. 1:**
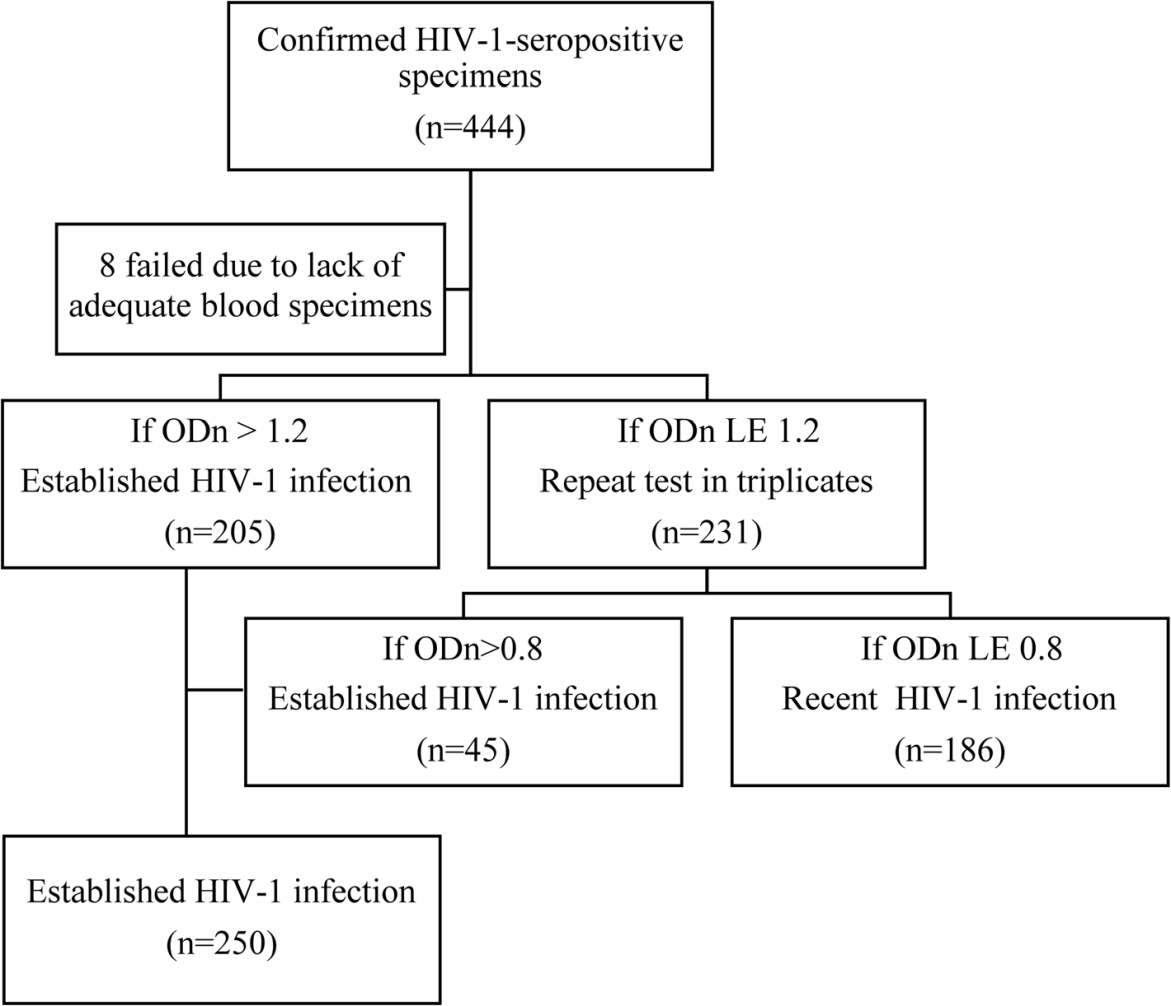
Algorithm for testing cross-sectional specimens to distinguish between established and recent HIV-1 infection. Abbreviations: OD = optical density

Samples of venous blood were collected from participants to diagnose HIV-1 antibodies. HIV-1 antibodies were detected using enzyme-linked immunosorbent assay [ELISA] (bioMerieux, Durham, NC, USA), and HIV-seropositive specimens were confirmed by western blotting [WB] (HIV Blot 2.2 WBTM, Genelabs Diagnostics, Singapore). The antibody test for HIV was conducted in provincial HIV laboratories of the Chinese Center for Disease Control and Prevention to which the seven study sites were affiliated. These WB-positive samples were tested using HIV-1 subtypes B, E, and D immunoglobulin G (IgG)-capture enzyme immunoassay [BED-CEIA] (Calypte Biomedical Corporation, Rockville, MD, USA) at the Key Laboratory of HIV/AIDS in Shenyang, China. Based on the measurement of HIV-1-specific IgG to total IgG after seroconversion, BED-CEIA distinguished between recent and established HIV-1 infections [26, 27]. To test cross-sectional specimens, we followed the algorithm shown in Fig. 1. The calibrator (CAL) and control (including high-positive control, low-positive control, and negative control) specimens were tested in triplicate on each plate, and median values were used to calculate the normalized OD (optical density) (ODn; ODn = specimen OD/calibrator OD). Specimens with initial ODn > 1.2 were classified as established infection. Specimens with initial ODn ≤1.2 were tested again in triplicate to confirm their ODn values using the median values of the three obtained values. During the retesting, if median ODn values were < 0.8, the specimens were considered to be recently infected [28].

### Statistical analyses

We used the chi-square test to determine the significance of differences in sociodemographics of the various subgroups of RD use. We then used the Cochran–Armitage trend test to analyze the association between different subgroups and sociodemographics. We calculated the prevalence, prevalence ratios and 95% confidence intervals of nine defined HIV-related high-risk behaviors for each subgroup of RD use. An alpha of 0.05 was considered statistically significant. The HIV incidence was estimated using a formula to adjust for sensitivity/specificity, and the time window to define recent HIV infection was up to 168 days. The formula and parameters were recommended by the Chinese Center for Disease Control and Prevention [29]. We then used multivariable logistic regression to determine the adjusted odds ratios (aOR) and respective 95% confidence intervals (CIs) of the various subgroups for recent or established HIV infection as defined by the BED-CEIA, adjusting for sociodemographics. Statistical analyses were carried out using SAS 9.2 (SAS Institute, Cary, NC, USA) and STATA 13.0 (Stata Corporation, College Station, TX, USA).

## Results

### Prevalence and patterns of use of multiple RDs

In total, 4496 MSM participated in our study. The mean age and standard deviation were 30.2 ± 5.6 years, and 1275 (28.4%) reported using RDs in the previous 6 months. Of these, 82.4% (*n* = 1050, 23.4% of all participants) used one type of RD (1DUs), 12.2% (*n* = 155, 3.4% of all participants) used two types of RDs (2DUs), and 5.5% (*n* = 70, 1.6% of all participants) used ≥3 types of RDs (3DUs). The prevalence of each unique RD use was as follows: poppers (25.9%), ecstasy (2.4%), ketamine (1.2%), amphetamine (0.6%), tramadol (0.4%), methamphetamine (3.8%) and codeine (1.9%).

**Fig. 2:**
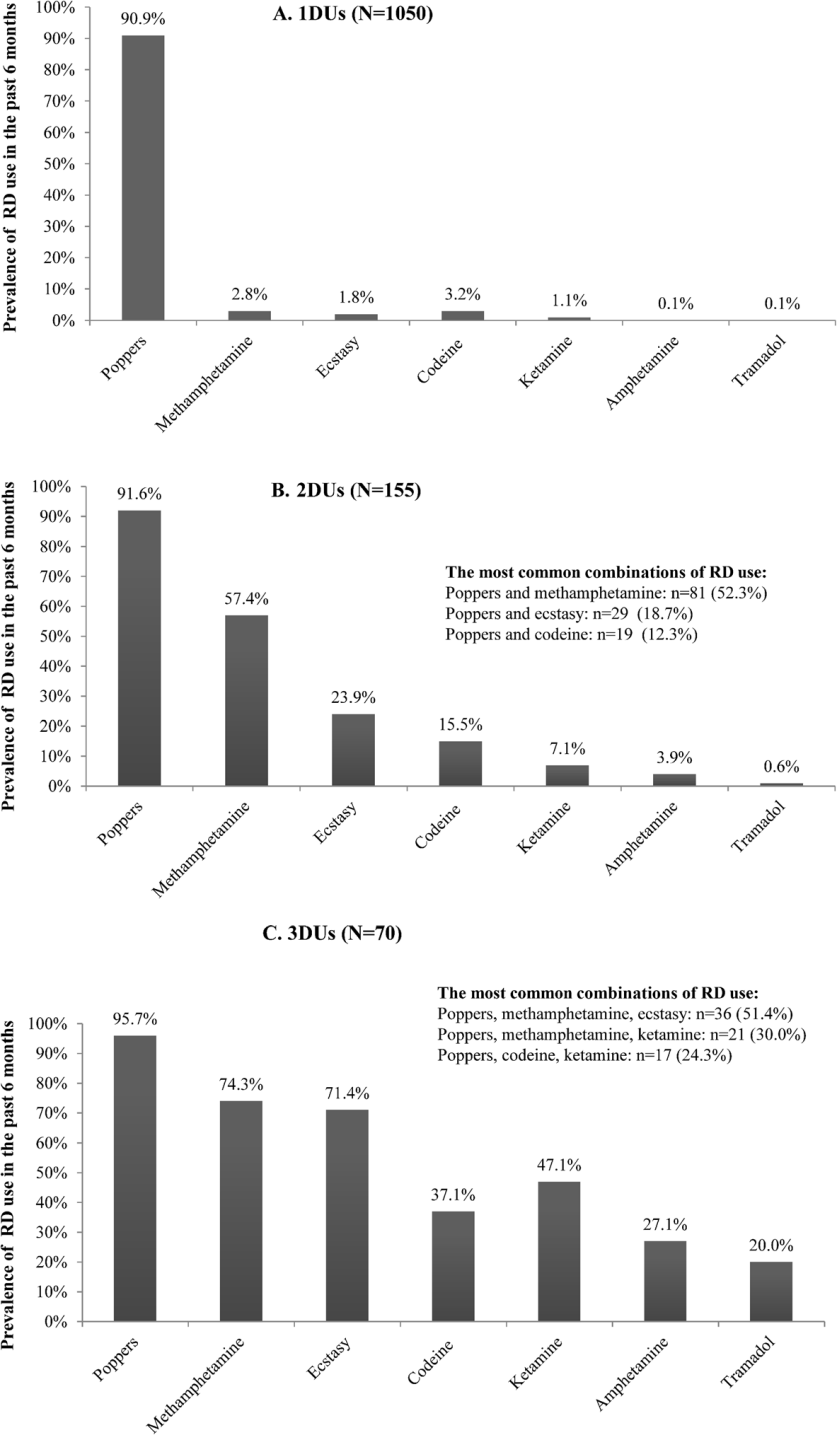
Prevalence of a specific RD use in the previous 6 months among different subgroups of RD use (*N* = 4496). 1DUs: One type of RD used in the previous 6 months; 2DUs: Two types of RDs used in the previous 6 months; 3DUs: Three or more types of RDs used in the previous 6 months. Out of the 4496 MSM participants, 1050 participants used one type of RD in the previous 6 months (Panel A), 155 used two types of RDs in the previous 6 months (Panel B), and 70 used three or more types of RDs in the previous 6 months (Panel C). The prevalence of the types of most common combinations of RD use are also listed above

Among 1DUs, most used poppers (90.9%), followed by codeine (3.2%) and methamphetamine (2.8%). Among 2DUs, 91.6% used poppers, 57.4% used methamphetamines, and 23.9% used ecstasy. Among 3DUs, 95.7% used poppers, 74.3% used methamphetamines, and 71.4% used ecstasy. Among all participants, 5.0% (*n* = 225, 17.6% of RD users) used two or more RDs (i.e., use of multiple types of RDs). These who used multiple types of RDs commonly took poppers accompanied by one or more types of other RDs (e.g., methamphetamine) (Fig. 2).

### Characteristics of the use of multiple types of RDs

We detected a higher prevalence of RDs use in the previous 6 months among MSM aged 26–30 years (vs. 18–25 and > 30 years; 39.0%), among participants who were internal migrants (vs. local cities; 30.1%), among those who never married (vs. married; 31.4%), for those who had a monthly income of ≥600 USD (vs. no income and 1–599 USD; 35.4%), among those who used several positions during anal intercourse (vs. top or bottom only; 32.2%), and among those with inadequate knowledge towards HIV-prevention (vs. adequate; 31.8%). The proportions of these sociodemographics associated with higher prevalence of RD use increased significantly as the number of RDs used in the previous 6 months increased *(P* < 0.05 for trend). Although participants who completed junior school or below had the lowest prevalence for the use of one type of RD in the previous 6 months (18.4%), the prevalence of use of two or more types of RDs in the previous 6 months was significantly higher than (8%) (Table 1).

**Table 1:** Distribution of sociodemographics among different subgroups of RD use (*N* = 4496)

	N	0DUs (*n* = 3221)	1DUs (n = 1050)	2DUs (*n* = 155)	3DUs (*n* = 70)	*P* for trend
**Age (years)**						
18–25	1617	1123	412	59	23	0.087
26–30	1181	721	375	63	22	< 0.001
> 30	1698	1377	263	33	25	< 0.001
**Residence**						
Local cities	1785	1327	385	45	28	0.002
Non-local cities	2711	1894	665	110	42	0.002
**Education**						
Junior school or below	916	674	169	45	28	0.17
High school	1201	877	269	37	18	0.227
College or above	2379	1670	612	73	24	0.972
**Marital status**						
Never married	3303	2266	860	129	48	< 0.001
Married	1193	955	190	26	22	< 0.001
**Occupation**						
Student	565	418	127	16	4	0.058
Non-student	3931	2803	923	139	66	0.058
**Monthly income (USD)**						
No income	769	591	146	25	7	0.001
1–599	2858	2069	658	94	37	0.042
≥ 600	869	561	246	36	26	< 0.001
**Primary sex position during AI**^a^						
Top	1395	1053	291	34	17	< 0.001
Bottom	988	725	222	31	10	0.067
Versatile	2022	1371	527	82	42	< 0.001
**Knowledge of HIV prevention**						
Inadequate	2730	1861	676	128	65	< 0.001
Adequate	1766	1360	374	27	5	< 0.001

*0DUs* No RDs used in the previous 6 months, *1DUs* One type of RD used in the previous 6 months, *2DUs* Two types of RDs used in the previous 6 months, *3DUs* Three or more types of RDs used in the previous 6 months, *AI* anal intercourse. The *P* for the trend of sociodemographics in different subgroups was determined using the Cochran–Armitage trend test a. Ninety-one participants did not respond to this variable on the questionnaire

### Characteristics of HIV-related high-risk behaviors

For almost all the HIV-related high-risk behaviors examined (e.g., seeking male sexual partners through the Internet in the previous 6 months, having group sex in the previous 6 months), the prevalence of HIV-related high-risk behavior was higher among those who used RDs in the previous 6 months than in those who did not. There was a significant association between HIV-related high-risk behavior and RD subgroup (*P* < 0.05 for all). In general, the prevalence of HIV-related high-risk behaviors increased as the number of RDs used in the previous 6 months increased (Table 2).

**Table 2:** Prevalence and adjusted prevalence ratios of HIV-related high-risk behaviors among different subgroups of RD use (*N* = 4496)

HIV-related high-risk behaviors	n/N	Prevalence	***P***	Adjusted prevalence ratios^a^
		% (95% CI)		(95% CI)
**Age of sexual debut with males ≤ 20 years**			*< 0.001*	
0DUs	1476/3219	45.9 (44.1–47.6)		1
1DUs	576/1049	54.9 (51.9–57.9)		1.2 (1.0–1.4)
2DUs	105/155	67.7 (60.3–75.2)		1.6 (1.1–2.4)
3DUs	45/70	64.3 (52.8–75.8)		1.6 (0.9–2.8)
**Seeking male sexual partners through the Internet in the previous 6 months**			*< 0.001*	
0DUs	2091/3221	64.9 (63.3–66.6)		1
1DUs	811/1050	77.2 (74.7–79.8)		1.6 (1.3–1.9)
2DUs	107/155	69.0 (61.7–76.4)		1.4 (0.9–2.0)
3DUs	46/70	65.7 (54.3–77.1)		1.5 (0.9–2.5)
**Had group sex in the previous 6 months**			*< 0.001*	
0DUs	112/3221	3.5 (2.8–4.1)		1
1DUs	55/1050	5.2 (3.9–6.6)		1.8 (1.2–2.5)
2DUs	6/155	3.9 (0.8–6.9)		2.3 (0.9–5.9)
3DUs	10/70	14.3 (5.9–22.7)		5.8 (2.4–14.4)
**Had more than two male sexual partners in the previous 6 months**			*< 0.001*	
0DUs	1184/3220	36.8 (35.1–38.4)		1
1DUs	490/1047	46.8 (43.8–49.8)		1.6 (1.3–1.8)
2DUs	77/155	49.7 (41.7–57.6)		1.3 (0.9–1.9)
3DUs	34/70	48.6 (36.6–60.6)		1.3 (0.8–2.1)
**Had commercial sex in the previous 6 months**			*< 0.001*	
0DUs	258/3221	8.0 (7.1–8.9)		1
1DUs	136/1050	13.0 (10.9–15.0)		1.2 (0.9–1.5)
2DUs	49/155	31.6 (24.2–39.0)		2.0 (1.3–3.0)
3DUs	16/70	22.9 (12.8–32.9)		1.3 (0.7–2.5)
**Had mucosally traumatic sex in the previous 6 months**			*0.002*	
0DUs	459/3221	14.3 (13.0–15.5)		1
1DUs	201/1050	19.1 (16.8–21.5)		1.4 (1.2–1.8)
2DUs	22/155	14.2 (8.6–19.7)		2.8 (1.5–5.1)
3DUs	13/70	18.6 (9.2–27.9)		1.9 (0.9–4.0)
**Had a condom break during AI in the previous 6 months**			*0.006*	
0DUs	255/3221	7.9 (7.0–8.8)		1
1DUs	109/1050	10.4 (8.5–12.2)		1.3 (1.0–1.7)
2DUs	14/155	9.0 (4.5–13.6)		2.3 (1.2–4.4)
3DUs	12/70	17.1 (8.1–26.2)		2.6 (1.2–5.6)
**Had STI-related symptom in the previous year**			*< 0.001*	
0DUs	169/3221	5.2 (4.5–6.0)		1
1DUs	81/1050	7.7 (6.1–9.3)		1.7 (1.3–2.3)
2DUs	15/154	9.7 (5.0–14.5)		2.4 (1.3–4.5)
3DUs	11/70	15.7 (7.0–24.5)		3.3 (1.6–6.8)
**Had non-Chinese male sexual partner in the previous 6 months**			*0.039*	
0DUs	30/2668	1.1 (0.7–1.5)		1
1DUs	17/867	2.0 (1.0–2.9)		2.4 (1.2–4.5)
2DUs	5/138	3.6 (0.5–6.8)		10.0 (3.1–31.9)
3DUs	1/51	2.0 (0.0–5.9)		6.6 (0.7–59.1)

*0DUs* No RDs used in the previous 6 months, *1DUs* One type of RD used in the previous 6 months, *2DUs* Two types of RDs used in the previous 6 months, *3DUs* Three or more types of RDs used in the previous 6 months, *AI* anal intercourse; Commercial sex: buying or selling sex services, including anal intercourse or oral intercourse; STI-related symptoms: abnormal pain or burning sensation upon micturition, urethra secretion, genital/anal skin damage, or hyperplasia; Mucosally traumatic sex: Mucosally traumatic sex means sexual intercourse especially anal intercourse sometimes may be rougher or last for longer and caused vagina, anus, or rectum mucosal trauma, which could facilitate HIV/STIs transmission. The table includes information only for participants who answered a specific question so that sample sizes may differ because of missing data. Unadjusted *P*-values were calculated by the chi-squared test. a: Adjusted prevalence ratios were calculated through multivariable log-binomial regression adjusting for the following sociodemographics: study site (Shanghai, Nanjing, Changsha, Zhengzhou, Ji’nan, Shenyang, and Kunming), age (18–25, 26–30, and > 30 years), residence (local cities and non-local cities), education (junior school or below, high school, and college or above), marital status (never married and married), occupation (student and non-student), monthly income (no income, 1–599, and ≥ 600 USD), primary sex position during AI (top, bottom, and versatile), and knowledge of HIV prevention (inadequate and adequate)

### Prevalence and incidence of HIV based on RD use

The HIV prevalence (13.7, 95% CI: 11.7–15.9 vs. 8.8, 95% CI: 7.8–9.8) and HIV incidence [13.1 infections per 100 person-years, 95% CI: 9.8–16.3 vs. 7.7 infections per 100 PY, 95% CI: 6.3–9.1] were higher among 1DUs than among participants who did not use RDs in the previous 6 months. By contrast, the HIV prevalence (8.4, 95% CI: 4.5–13.9; 7.1, 95% CI: 2.4–15.9) and HIV incidence (9.7 infections per 100 PY, 95% CI: 2.5–16.9; 2.4 infections per 100 PY, 95% CI: − 2.3–7.0) in 2DUs and 3DUs were not significantly higher compared to those who did not use RDs or used one RD in the previous 6 months (Table 3).

**Table 3:** HIV prevalence and BED-CEIA-based HIV incidence among Chinese MSM participants (*N* = 4496)

	Total	HIV infection	HIV recent infection	HIV established infection	HIV prevalence (%) (95% CI)^a^	HIV incidence (per 100 PY) (95% CI)^b^
**Subgroups of RD use**	4496	444	186	250	9.9 (9.0–10.8)	8.9 (7.6–10.2)
0DUs	3221	282	116	161	8.8 (7.8–9.8)	7.7 (6.3–9.1)
1DUs	1050	144	62	79	13.7 (11.7–15.9)	13.1 (9.8–16.3)
2DUs	155	13	7	6	8.4 (4.5–13.9)	9.7 (2.5–16.9)
3DUs	70	5	1	4	7.1 (2.4–15.9)	2.4 (−2.3 to 7.0)

*0DUs* No RDs used in the previous 6 months, *1DUs* One type of RD used in the previous 6 months, *2DUs* Two types of RDs used in the previous 6 months, *3DUs* Three or more types of RDs used in the previous 6 months. ^a^HIV prevalence was calculated from all HIV infections (i.e., recent and established) diagnosed through the study. The BED-CEIA could not be conducted on samples from eight HIV antibody-positive participants because of insufficientblood specimens. ^b^The HIV incidence determined using the BED-CEIA was then adjusted using the sensitivity and specificity adjustment formula and parameters recommended by the Chinese Center for Disease Control and Prevention

### Association between RD use and HIV infection

After adjusting for sociodemographics, 1DUs had higher odds of established HIV infection (aOR = 2.1, 95% CI: 1.5–2.8) and higher odds of recent HIV infection (aOR = 2.2, 95% CI: 1.5–3.0) than those who did not use RDs. 2DUs also had higher odds of recent HIV infection (aOR = 2.3, 95% CI: 1.0–5.2) than those who did not use RDs. In contrast, 2DUs and 3DUs did not have significantly higher odds of established HIV infection than those who did not use RDs (Table 4).

**Table 4:** Adjusted odds ratios for recent or established HIV infections stratified by RD use subgroup (*N* = 4488)^a^

Subgroups of RD use	Total	Recent HIV infection (***N*** = 186)			Established HIV infection (***N*** = 250)		
		n (%)	Adjusted model^b^	*P*	n (%)	Adjusted model^b^	*P*
			aOR (95% CI)			aOR (95% CI)	
0DUs	3216	116 (3.6)	1	–	161 (5.0)	1	–
1DUs	1047	62 (5.9)	2.2 (1.5–3.0)	< 0.001	79 (7.5)	2.1 (1.5–2.8)	< 0.001
2DUs	155	7 (4.5)	2.3 (1.0–5.2)	0.054	6 (3.9)	1.4 (0.6–3.4)	0.433
3DUs	70	1 (1.4)	0.8 (0.1–5.8)	0.809	4 (5.7)	1.9 (0.6–5.4)	0.247

*0DUs* No RDs used in the previous 6 months, *1DUs* One type of RD used in the previous 6 months, *2DUs* Two types of RDs used in the previous 6 months, *3DUs* Three or more types of RDs used in the previous 6 months. ^a^Recent and established infections were determined using the BED-CEIA. The BED-CEIA could not be conducted on samples from eight HIV antibody-positive participants because of insufficient blood specimens. ^b^Adjusted odds ratios (aOR) and the corresponding 95% CI were derived through multivariable logistic regression analysis with adjustment for the following social demographics: study site (Shanghai, Nanjing, Changsha, Zhengzhou, Ji’nan, Shenyang, and Kunming), age (18–25, 26–30, and > 30 years), residence (local cities and non-local cities), education (junior school or below, high school, and college or above), marital status (never married and married), occupation (student and non-student), monthly income (no income, 1–599, and ≥ 600 USD), primary position during AI (top, bottom, and versatile), and knowledge of HIV prevention (inadequate and adequate)

## Discussion

We examined patterns of use of RDs among Chinese MSM and the impact of such RD use on HIV-related high-risk behaviors and HIV acquisition. We found that the prevalence of use of multiple RDs among Chinese MSM (5.0%) was lower than the reported prevalence of use of multiple RDs among MSM in western high-income countries (11.8%) [2]. Chinese MSM who used multiple RDs frequently used poppers along with one or more types of other RDs. As the number of RDs used in the previous 6 months increased, the prevalence of various HIV-related high-risk behaviors also increased. The odds of recent HIV infection were higher among those who used RDs in the previous 6 months than among those who did not. These data suggest that those who use multiple RDs are substantially more likely to engage in risky sexual behaviors and may have a higher risk of HIV acquisition.

Our findings expand the understanding of the RD use in MSM in a low-to-middle income country and the relationship of multiple RD use with HIV infection. Previous studies showed correlations between RD use and HIV-related high-risk behaviors among MSM [6, 30]. Our study confirmed those results and found that the increase in the number of RDs used was associated with an increase in the prevalence of HIV-related high-risk behaviors. A similar finding was documented among HIV-positive MSM in the United Kingdom [16]; however, until now, this relationship has not been examined in a low-to-middle income country. In contrast with patterns of use of multiple RDs among MSM in high-income countries who frequently use [16, 17, 31], Chinese MSM frequently use poppers. We found that 90% of MSM used poppers, and those who used multiple RDs frequently used poppers and one or more types of other RDs (i.e., methamphetamine, ecstasy, codeine, or ketamine). Poppers are physiologically active substances that facilitate and enhance anal intercourse [2, 12]. They do not directly affect mental function or decision-making [32]. However, when poppers were used with other psychoactive drugs [2] such as methamphetamine, ecstasy, or ketamine, the prevalence of HIV-related high-risk behaviors increased significantly (Table 2). Most psychoactive drugs are stimulants and, thus, can alter the mental state [15], cause loss of muscle control [15], enhance sexual desires/sexual functions, and affect risk perception and decision-making [10, 11]. If psychoactive drugs are used simultaneously with poppers (which relax the anal-sphincter muscles and reduce pain), MSM can experience more serious sexual disinhibition and, thus, anal intercourse may be more robust or last longer, leading to increased risk of HIV infection [32]. This pattern of use of multiple RDs is widespread among Chinese MSM and was associated with more HIV-related high-risk behaviors in our study. Scholars also found that using poppers with methamphetamine or amphetamine was associated with more unprotected sex acts [33] and, hence, was linked to a higher risk of HIV seroconversion [34, 35].

The proportion of HIV-related high-risk behaviors increased as the number of RDs used in the previous 6 months increased. However, possible relationship between HIV-related high-risk behaviors, the number of RDs used, and HIV infections are not clear. When we subdivided diagnosed HIV infections into recent and established HIV infections based on the BED-CEIA, we found a significant relationship between higher odds of recent HIV infection and one type of RD used or two types of RD used compared with no RD used in the previous 6 months. While we did not find a significant relationship between higher odds of recent HIV infection and 3DUs compared to those who did not use RDs, this finding could have been due to our small sample size and low statistical power to detect the differences in our aOR estimates. In contrast, we found a significant relationship between established HIV infection and one type of RD used in the previous 6 months compared with no RD use. This result suggests that RDs used in the previous 6 months were associated in particular with recent HIV infection. This may have been because those use multiple RDs are less risk-averse and more likely to engage in more risky sexual behaviors and, thus, may have higher risk of new HIV infections. [41, 42] Several longitudinal studies found similar associations between the use of multiple RDs and recent HIV seroconversion [18, 36]. Newly infected individuals have high levels of viral load in plasma and few pronounced symptoms and are highly infectious; However, identifying newly infected individuals is difficult [37, 38]. MSM in this HIV seroconversion period have been found to participate frequently in HIV-related high-risk behaviors [38]. Thus, those who use multiple RDs likely take more sexual risks and therefore may have an increased risk of secondary HIV transmission.

Although the prevalence of use of multiple RDs among Chinese MSM in our study was lower than the prevalence among MSM reported in high-income countries (5.0% vs. 11.8%) [2], the actual prevalence of use of multiple RDs of Chinese MSM may have been underestimated. First, most RDs are illegal in China, so participants may have been afraid of the repercussions of answering honestly about their use of multiple RDs. To address these fears, we used an anonymous survey to minimize this social desirability bias. Second, we investigated only the seven most commonly used RDs among Chinese MSM. There are many more RD used by MSM that we did not address in this study.

We elucidated the characteristics and possible mechanisms of HIV transmission among Chinese MSM who use RDs. We undertook this study in the hope of developing targeted interventions to address the HIV epidemic. We found that participants aged 26–30 years had a higher prevalence of overall RD use in the previous 6 months (39.0%) and use of multiple RDs in the previous 6 months (7.2%) compared with any other age group examined. The number of identified HIV/AIDS cases among MSM aged 26–30 years in China increased rapidly from 2007 to 2015 [39]. Mao and colleagues found a high HIV incidence (~ 9 infections per 100 PY) among Chinese MSM aged 26–30 years [40]. RD use was associated with behaviors that increase the risk of HIV, suggesting that tailored methods reducing RD use implemented among Chinese MSM 26–30 years old could reduce the risk of HIV acquisition. We also found an association between inadequate knowledge of HIV prevention and an increase in the number of RDs used in the previous 6 months, suggesting that strategies focused on increasing positive attitude towards prevention of HIV infection should be targeted at those who use multiple RDs. Finally, many Chinese MSM in our study used poppers, which may have been due to the ease of buying this drug as it is legal in China [19]. We suggest that the Chinese government restrict the sale of poppers to reduce RD use and decrease the effect of RD use on HIV transmission.

One limitation or our study was the few participants who self-reported using multiple types of RDs (5.0%). The few participants in this category likely reduced the power of our study to detect differences in the different drug use sub-groups. We also did not collect the link-to-care details of the participants diagnosed with HIV in this study. However, China has already established a standard procedure for individuals diagnosed with HIV to obtain free antiretroviral therapy (ART) treatment and follow-up testing. Once a person was diagnosed with HIV in this study, the local Chinese Center for Disease Control and Prevention would refer him to the local infectious disease hospital to obtain free ART treatment. The government would allocate ART treatment medications and pay for the costs of follow-up tests.

## Conclusions

Chinese MSM who used multiple RDs frequently used poppers along with one or more types of other RDs. The proportion of HIV-related high-risk behaviors increased as the number of RD used in the previous 6 months increased. Using one or two types of RDs was associated with increased odds of recent HIV infection among Chinese MSM compared to the odds of those who did not use RDs. Therefore, the use of several types of RDs is likely associated with new HIV infections. Strategies focusing on decreasing RD use among Chinese MSM and increased governmental control of RDs could reduce drug use and mitigate the HIV epidemic in China.

## Supplementary Information

**Additional file 1.** Questionnaire developed for this study

## Data Availability

Data on this research is available with the corresponding author.

## Abbreviations

MSM: Men who have sex with men
HIV: Human immunodeficiency virus
RDs: Recreational drugs
HIV-HRBs: HIV high-risk behaviors
CLS: Condomless sex
WB: Western blotting
BED-CEIA: Immunoglobulin G (IgG)-capture BED-enzyme immunoassay
CIs: Confidence intervals
AORs: Adjusted odds ratios
ORs: Odds ratios
1DUs: Users of one type of RD
2DUs: Users of two types of RDs
3DUs: Users of three or more types RDs
