# Supplementary material for: Use of multiple recreational drugs is associated with new HIV infections among men who have sex with men in China: a multicenter cross-sectional survey
Source: BMC Public Health. 2021 Feb 15;21:354. doi: 10.1186/s12889-021-10223-y (PMC7885486; doi:10.1186/s12889-021-10223-y)
Supplement: Supplementary file 1 — Additional file 1. Questionnaire developed for this study [file 12889_2021_10223_MOESM1_ESM.docx]

**QUESTIONNAIRE**

Age

1. 18-25 2.26-30 3.＞30

Had mucosally-traumatic sex in the P6M

1.Yes 2.No

The test result of BED-CEIA

1.positive

2.negitive

Is it possible for a person who looks healthy to carry HIV?

1.possible

2.impossible

3.I don’t know

Is it possible to be infected through transfusion of blood or blood products with HIV?

1.possible

2.impossible

3.I don’t know

Is it possible to be infected through sharing needles with HIV-infected persons or AIDS patients? 1.possible

2.impossible

3.I don’t know

Can proper use of condoms in each sexual activity reduce the risk of HIV transmission?

1.possible

2.impossible

3.I don’t know

Maintaining only having sex with a single HIV-uninfected sexual partner whether can reduce the risk of HIV transmission?

1.possible

2.impossible

3.I don’t know

Can an HIV-infected pregnant woman can transmit HIV to her child?

1.possible

2.impossible

3.I don’t know

Is it possible to be infected through eating with HIV-infected persons or AIDS patients?

1.possible

2.impossible

3.I don’t know

Is it possible to be infected through mosquito bites?

1.possible

2.impossible

3.I don’t know

If you know or suspect that your partner has AIDS, will you stop having sex with him?

1.possible

2.impossible

3.I don’t know

Had commercial sex in the P6M

1.Yes

2.No

Had condom break during AI in the P6M

1.Yes

2.No

Had non-Chinese male sexual partners in the P6M

1.Yes

2.No

Primary sex position during AI

1.Top

2.Bottom

3.Versatile

Recreational drug use

1.Yes

0.No

Education

1.Junior school or below

2.High school

3.College or above

Had group sex in the P6M

1.Yes

0.No

Have you used Rush in the last 6 months

1.Yes

0.No

Have you used ecstasy in the last 6 months

1.Yes

0.No

Have you used ketamine in the last 6 months

1.Yes

0.No

Have you used amphetamine in the last 6 months

1.Yes

0.No

Have you used tramadol in the last 6 months

1.Yes

0.No

Have you used methamphetamine in the last 6 months

1.Yes

0.No

Have you used codeine in the last 6 months

1.Yes

0.No

HIV infection

1.positive

2.negitive

HIV prevention knowledge

1.Inadequate

2.Adequate

Had STIs-related symptom in the past year

1.Yes

2.No

Monthly income (USD)

1. No income

2.1-599

3.≥600

Age of sexual debut with males<=20

1.Yes

2.No

Marital status

1.Never married

0.Married

Had more than 2 male sexual partners in the P6M

1.Yes

2.No

Occupation

1.Student

2.Non-student

ID number

Poly drug use

0. No drugs used in the P6M

1. One type of drug used in the P6M

2. Two type of drugs used in the P6M

3. Three type of drugs used in the P6M

Residence

1. Local cities

2. Non-local cities

Age

Sought male sexual partners through the Internet in the P6M

1.Yes

2.No

Study site

2.kunming

3.shenyang

4. jinan

5.changsha

6. zhengzhou

8.nanjing

9.shanghai
